# Supplementary material for: Research on multi-condition optimization of centrifugal compressor impeller meridian profile
Source: PLoS One. 2025 Feb 11;20(2):e0318783. doi: 10.1371/journal.pone.0318783 (PMC11813096; doi:10.1371/journal.pone.0318783)
Supplement: Attachment — Submitted filename: Response to Reviewers.docx [file pone.0318783.s001.docx]

**“Research on Multi-condition Optimization of Centrifugal Compressor Impeller Meridian Profile”**

**Revision Explanation**

Respect the editor. Hello, thank the editorial department and peer review experts for their meticulous and patient review. This paper modification work aims to further improve the content of the paper, ensuring it conforms to the norms and requirements of academic papers. The following is the detailed content of this modification:

Reviewer #1:

**1)**There are problems such as grammatical errors, unclear ideation and improper use of the article, so it is necessary to improve the full text language.

**Response:** Grammar has been thoroughly combed, diction screened, and the entire text has been embellished.

**2）**The consistency of terminology must be ensured, for example, the term "meridional contour" should be used uniformly in the abstract and the whole paper.

**Response:** A comprehensive verification and adjustment process has been carried out to eliminate possible confusion, such as synonyms, and to ensure consistency and standardization in the use of terminology.

**3)**When the impeller hub rim changes, the extension mode of the blade shape should be elaborated.

**Response:** In the first paragraph of subsection 3.1 of the paper, a targeted supplementary explanation has been made: it is made clear that the design scheme adopted in this paper focuses on the rim and hub profiles, and in response to changes in the impeller hub rims, the overall extension of the blades and the degree of curvature is maintained in line with the prototype scheme, so as to guarantee the design consistency and functionality of the adaption.

**4)**Explain why you chose the single channel calculation and why you did not use other calculation methods.

**Response:** As detailed in the second paragraph of subsection 2.3 of the paper, the single-channel calculation is chosen to utilize the impeller periodic boundary conditions, which can reduce the amount of calculation and avoid the waste of resources.

**5)**Within a certain speed range, compressor characteristics should be similar. If so, is it more practical to highlight the coordination of multiple operating conditions (not less than 3 points) at the design speed? On the contrary, it is necessary to emphasize the reasons for the obvious difference in characteristics when the speed changes.

**Response:** At the penultimate paragraph of chapter 1, an explanation is added to illustrate the significance of optimization at multiple rotational speeds; as the impeller speed increases, the blade tip speed increases, generating centrifugal force and compression effects are also enhanced. At low speeds, the airflow is under-energized and the pressure ratio and efficiency are lower, while at higher speeds, although the pressure ratio increases, the efficiency decreases due to increased flow losses.

Reviewer #2:

**1)**The article is unclear, lacks logic, and has some sentences with incorrect expressions. From the abstract, it can be seen that the author of this article intends to focus on using the NSGA-III method, but there are few references to the NSGA-III method throughout the full text, and there is not a single reference cited in the entire third section.

**Response:** The article has been sorted out and checked sentence by sentence and paragraph by paragraph, focusing on the optimization and adjustment of the logical chain, and correcting the sentences that are wrongly expressed, so as to make the connection between the lines smoother and the meaning more precise. In the application of the method, only the NSGA-III method is used as a tool for optimization, and for the problem of unclear expression, detailed references have been added in Chapter 3, aiming to make the application of the method more fully based on the theoretical support, which is in line with the requirements of academic norms.

The editorial team is kindly requested to consider revising the notes and, if anything, to give the opportunity for further revisions.

Ning Yu, Xiaohan Yu, Zhi Cai, Xinle Yang, Liyong Tian, Song Li
